# Engagement and outcomes of cancer patients referred to a tobacco cessation program at a National Cancer Institute‐designated cancer center

**DOI:** 10.1002/cam4.5423

**Published:** 2022-11-29

**Authors:** Sarah A. Westergaard, Manali Rupji, Lauren E. Franklin, Madhusmita Behera, Suresh S. Ramalingam, Kristin A. Higgins

**Affiliations:** ^1^ Winship Cancer Institute of Emory University Atlanta Georgia USA

**Keywords:** C3I, cancer, NCI, tobacco cessation

## Abstract

**Introduction:**

Tobacco cessation is a critical but challenging intervention for cancer patients. Our National Cancer Institute‐designated Comprehensive Cancer Center instituted a tobacco cessation program in 2019. This manuscript reports on the first 2 years of our experience.

**Methods:**

Patients were referred to the program by their care team, and a certified tobacco treatment specialist contacted patients remotely and provided behavioral therapy and coordinated pharmacotherapy. We retrospectively captured data from patients with a cancer diagnosis referred to the tobacco cessation program. Univariate and multivariable logistic regression analyses with the backward elimination approach were performed to determine factors associated with patient acceptance of referral to the tobacco cessation program. Tobacco cessation rates after referral to the program were also captured.

**Results:**

Between July 2019 and August 2021, 194 patients were referred to the tobacco cessation program. Of the 194 patients referred, 93 agreed to enroll in the tobacco cessation program (47.9%), of which 84 requested pharmacotherapy (90.3%). Twenty‐four were able to cease tobacco use (25.8%). Only 7 patients out of the 101 patients (6.9%) who declined cessation services were successful (*p* < 0.001). On univariate logistic regression, race (*p* = 0.027) and marital status (*p* = 0.020) were associated with referral acceptance. On multivariable analysis, single patients (odds ratio [OR] = 0.33) and Caucasian patients (OR = 0.43) were less likely to accept a referral.

**Conclusions:**

Access to tobacco cessation services is a critical component of comprehensive cancer care. Our experience highlights the need to understand patient‐specific factors associated with engagement with a tobacco cessation program during cancer treatment. The use of pharmacotherapy is also a critical component of successful tobacco cessation.

## Graphical abstract

At a National Cancer Institute‐designated cancer center, a tobacco cessation program was implemented, and under half of cancer patients engaged in the program. Single patients and Caucasian patients were less likely to engage with the program. Further efforts are needed to increase engagement of patients with tobacco cessation programs as we found higher tobacco cessation rates with program participation.

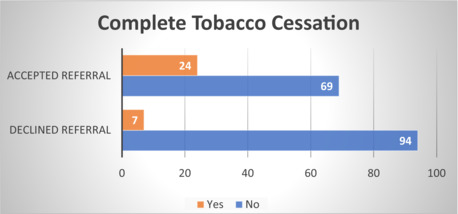

## 1 INTRODUCTION

According to the Center for Disease Control (CDC), cigarette smoking is responsible for about 1 in 5 deaths in the United States (US).^1^ Although the reviewer reports smoking is responsible for 1/5 of *early* deaths, the report of the Surgeon General directly states that “cigarette smoking is responsible for more than 480,000 deaths per year in the United States… This is about one in five deaths annually, or 1300 deaths every day.” It does specify early deaths in particular. It does however note that “smokers die 10 years earlier than nonsmokers.” Fortunately, the rate of smoking has been on the decline for decades. Whereas 20% of adults in the US were current smokers in 2005, only 14% of adults were current smokers in 2019.^2^ However, cigarette smoking is still the number one cause of preventable disease, disability, and death in the US.^1^ Tobacco products incur a physical, societal, and financial burden amounting to hundreds of billions of dollars per year due to medical care costs and loss of work productivity.^3^, ^4^ While cigarette use has decreased, there has been a rise in the use of electronic nicotine delivery systems, termed “e‐cigarettes,” leading the U.S. surgeon general to declare a youth e‐cigarette “epidemic” in 2018.^5^ Marketed as being a safer alternative to cigarettes, the aerosols that are produced also potentially contain toxic substances, including known carcinogens^6^, ^7^

It is imperative to drive tobacco product use to even lower rates. Outside of the devastating impact of tobacco products on pulmonary and cardiovascular function, there is a well‐known association between tobacco use and the development of at least 12 different types of cancer.^8^ Tobacco products contain numerous carcinogens that cause DNA damage, leading to mutations in oncogenes and tumor‐suppressor genes, which can result in unregulated growth leading to cancer. Tobacco is the leading preventable cause of cancer, with 30% of cancer deaths caused by tobacco products.^9^, ^10^, ^11^ While nicotine itself is not a carcinogen, its addictive properties make it difficult for tobacco users to quit, increasing their exposure to carcinogens over time.

Since tobacco products were first declared to be hazardous in 1964, there has been an impressive effort to develop public health initiatives and increase education on the deleterious effects of tobacco use.^12^ Despite this, tobacco use remains prevalent, especially in certain subgroups of the population. While the majority of smokers report a desire to quit, only about 7% were able to quit smoking in the past year.^13^ At the time of a cancer diagnosis, around a quarter of patients are currently using tobacco products.^14^ Even after a cancer diagnosis, when patients report high motivation to quit using tobacco products, less than half are successful in tobacco cessation.^15^ Close to one in seven cancer survivors are also current cigarette smokers.^16^

Patient‐identified barriers to tobacco cessation include environmental factors, concern for weight gain, perceived difficulty, lack of desire, cost of nicotine replacement, and lack of support from healthcare providers.^17^ Cancer patients face additional barriers to tobacco cessation, including increased distress and a perceived lack of benefit.^18^ Therefore, providers should be cognizant of the unique challenges of cancer patients and tailor interventions accordingly.^19^

Tobacco cessation is of the utmost importance in cancer patients because continued tobacco use is associated with inferior response to treatment, compromising oncologic outcomes, as well as increased risk of additional primary cancers.^18^, ^20^ Tobacco cessation services are essential to comprehensive cancer care and should be offered to all patients with a cancer diagnosis. Our single‐institution cancer center sought to create and implement a tobacco cessation program within our cancer center. This manuscript depicts the patient population referred to our program over the first 2‐year period, as well as quit rates, rates of pharmacotherapy usage, and patient factors associated with engagement with cessation services.

## 2 METHODS

**FIGURE 1:**
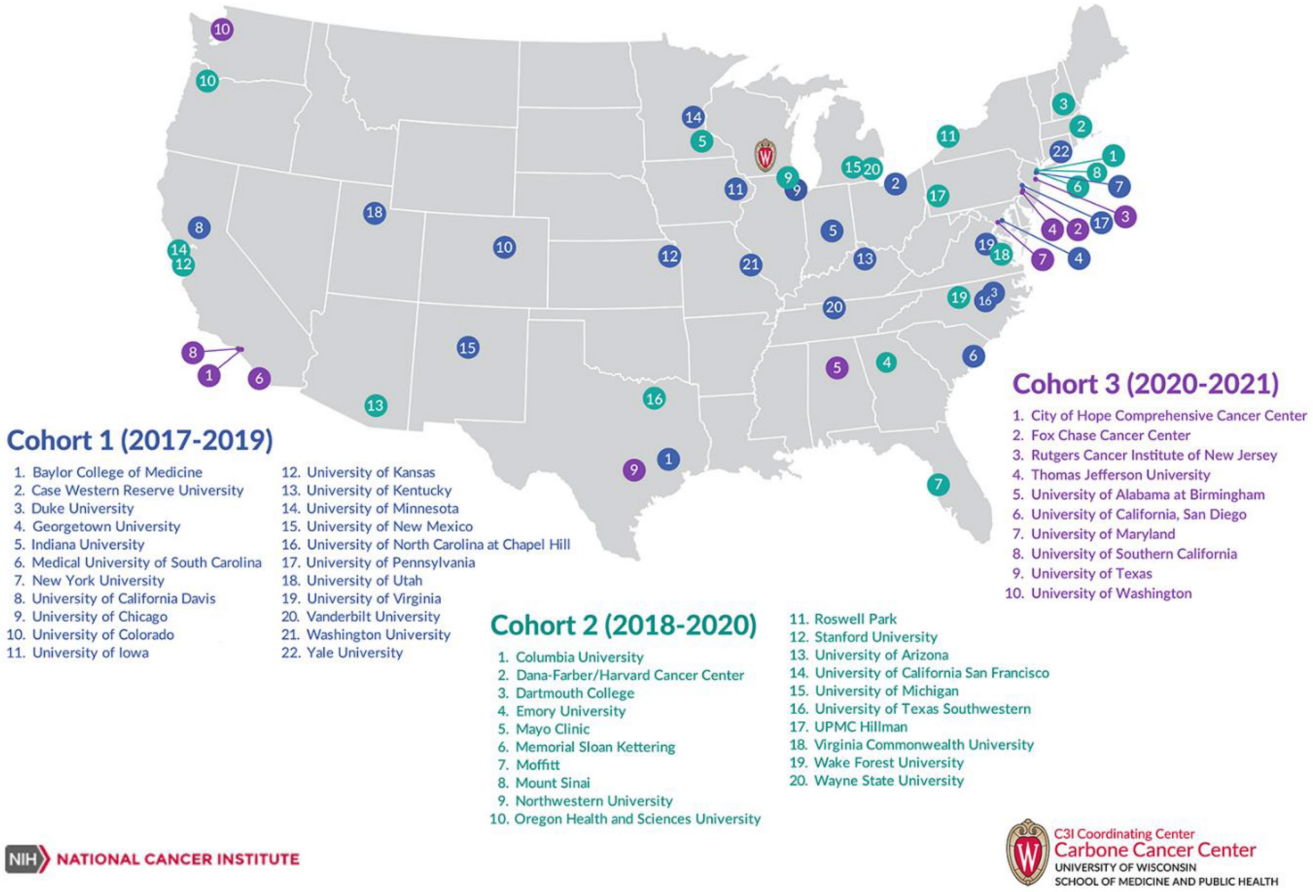
Map of National Cancer Institute‐designated Comprehensive Cancer Centers that received funding from the National Cancer Institute's Cancer Center Cessation Initiative (C3I), which provides funding for cancer centers to establish tobacco cessation programs nationwide. The Cancer Center Cessation Initiative Coordinating Center and Expert Advisory Panel. (2021). Introduction to the Cancer Center Cessation Initiative Working Groups: Improving Oncology Care and Outcomes by Including Tobacco Treatment, Journal of the National Comprehensive Cancer Network, 19(Suppl_1), S1‐S3.

Our NCI‐designated comprehensive cancer center received funding through a supplemental grant to our comprehensive cancer center grant, specifically the Cancer Center Cessation Initiative (C3I) funding mechanism, which was designed to provide funding for cancer centers to establish tobacco cessation programs. This funding mechanism was established in 2018 and will complete in 2022. Our cancer center received funding in the second cohort of institutions (three cohorts total), in 2018 (Figure 1). After approximately 12 months of planning, we launched our program in July 2019.

Cancer patients using tobacco products were identified by providers during office visits and referred to the program through the electronic health record. Patients were also able to self‐refer to the program, and promotional materials were available at all outpatient clinics. Patients were included in the study if they were current smokers and expressed an interest in a referral to the Winship Cancer Center tobacco cessation program between July 2019 and August 2021. They were excluded if they were noncurrent smokers or did not wish to be referred to the program. The program consists of entirely remote cessation services provided by a certified tobacco cessation specialist that was free of charge to the patient.

**FIGURE 2:**
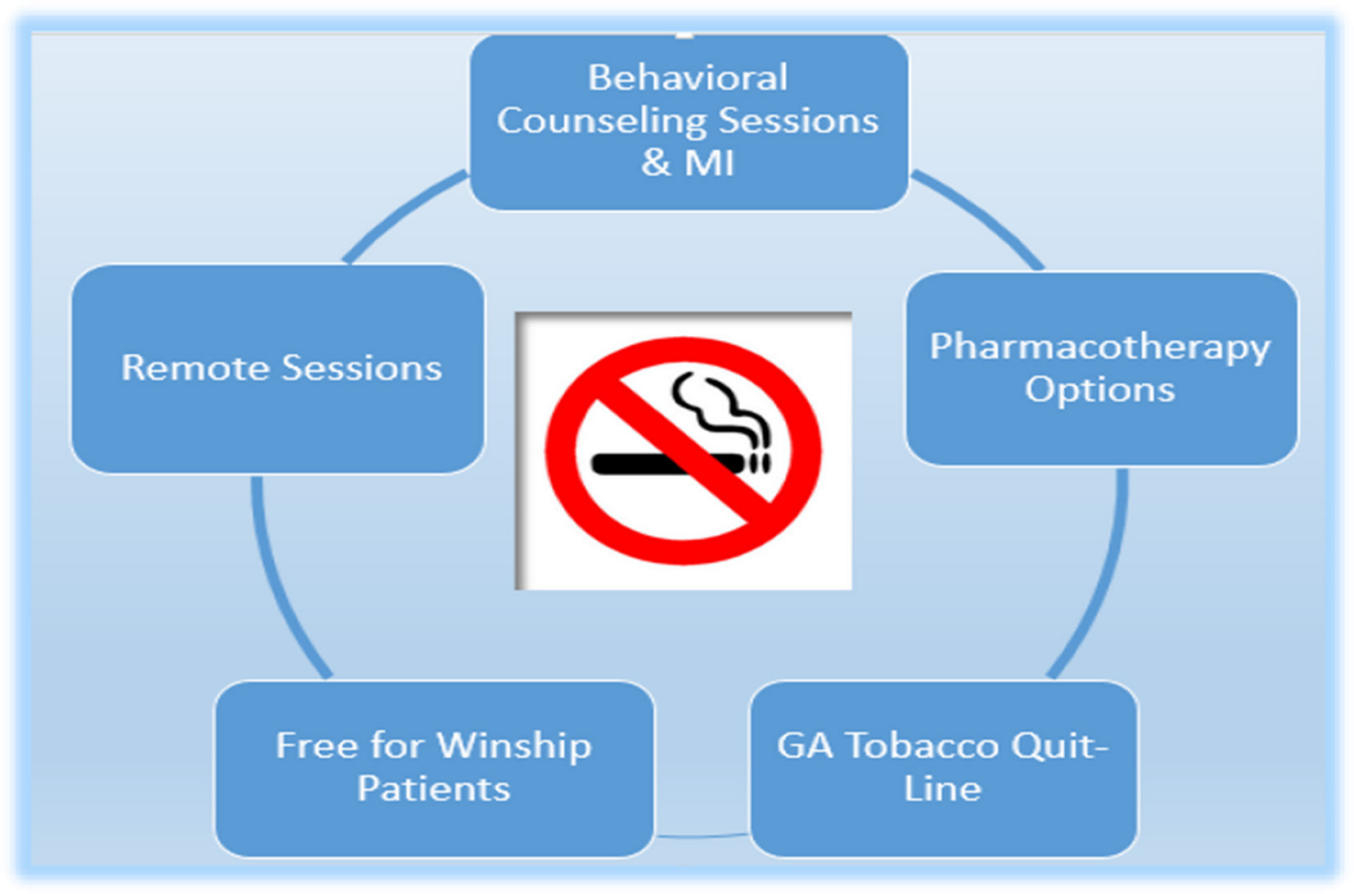
Once a patient was referred to the tobacco cessation program, a certified tobacco cessation specialist contacted patients remotely free of charge and provided behavioral counseling sessional and motivation interviewing, as well as screened for pharmacotherapy and made referrals to the Georgia tobacco quitline.

After the referral was placed by the care team, the tobacco treatment specialist proactivity reached out to the patient and scheduled an initial assessment. The assessment included details about their current smoking status as well as questions about their smoking history. They were educated on the benefits of cessation medication and screened for any contraindications that they may have. A clinical message was sent to their referring provider with pharmacotherapy details and patients retrieved their medication through their pharmacy. They were provided with the option to choose cessation counseling with the Georgia Tobacco Quit Line or through the program's Smoke Free Text Messaging resource. The assessment concluded with creating a personalized quit plan that was reviewed with the patient during follow‐up calls (Figure 2). We sought to investigate if there were any associations between specific patient characteristics and the likelihood of referral acceptance. After obtaining Institutional Review Board approval, 194 patients referred to the program between July 2019 and August 2021 were retrospectively identified. There were more smokers identified, but they were not interested in a referral and are not included in this analysis.

Demographic data were captured for all these patients, including age, gender, race, education level, religious affiliation, marital status, and the median income of their documented ZIP code, as well as their insurance type and any documented mental health diagnoses. Rates of tobacco cessation were determined by our tobacco cessation specialist and her interactions with the patient, and these rates were captured in the chart reviews. Of note, tobacco cessation was not confirmed with biochemical verification.

First, a univariate logistic regression was performed with acceptance to the referral program as the event of interest. We used the term “univariate” association to describe the association of each independent variable with the outcome (dependent variable) to distinguish it from multivariable analysis in which two or more independent variables are assessed in relation to a dependent outcome. In this context, the term univariate appropriately replaces the term “bivariate.” Then, a multivariable analysis was performed using the backward elimination approach to identify factors associated with referral acceptance. Education level and religious affiliation were excluded from multivariable analyses given a large amount of missing data.

Association of those on cessation program and cessation of tobacco use was performed using Pearson's chi‐square test. Out of the 194 participants, only 8 self‐referred. Given the small number, any analyses evaluating the association of self‐referral with tobacco cessation success would be underpowered, so none were performed. Statistical analysis was performed using SAS 9.4 (SAS Institute, Inc.), and statistical significance was assessed at the 0.05 level.

## 3 RESULTS

From July 2019 to August 2021, 194 patients were referred for tobacco cessation services by their oncology care teams. Ninety‐three agreed to participate in the program (47.9%). Of 194 patients, 106 were male (54.6%) and 88 were female (45.4%). The median age was 61 years old (range 21–84). The majority were Caucasian (104, 53.6%) followed by African Americans (75, 38.7%), Hispanic (2, 1%), Asian (1, 0.5%), and unknown race (12, 6.2%). The majority (81, 43.5%) were married, 65 (34.9%) were single, 25 (13.4%) were divorced, and 15 (7.7%) were widowed. Most patients identified as having a religious affiliation (76, 39.2%), compared with 65 who did not identify as being religious (33.5%). A large number of patients, 53, had unknown religious affiliations.

The education level was unavailable for 122 patients. Thirty‐five patients had an education level of high school or less (48.6%), and 37 went to college or graduate school for at least some period (51.4%). Forty‐six patients (23.4%) had a documented mental health diagnosis. Most patients had private insurance (110, 56.7%), followed by Medicare (50, 25.8%), or were uninsured or had Medicaid (34, 17.5%).

Most patients (138, 71.9%) lived in an area with a median annual income of $40,000–$75,000, followed by over $75,000 (36, 18.8%) and less than $40,000 (18, 9.4%). Sixty‐nine patients had a lung cancer diagnosis (40.1%). Thirty patients had a head and neck cancer diagnosis (17.4%), and the remainder of the patients had other cancer diagnoses (73, 42.4%) (Table 1).

**TABLE 1:** Demographic and clinical data of patients referred to a tobacco cessation program by their cancer care provider between July 2019 and August 2021

Variable	Level	*N* (%) = 194
Gender	Male	106 (54.6)
	Female	88 (45.4)
Race	Caucasian	104 (57.1)
	Non‐Caucasian	78 (42.9)
	Missing	12
Insurance	Private	110 (56.7)
	Medicare	50 (25.8)
	Medicaid/uninsured	34 (17.5)
Marital status	Married	81 (43.5)
	Single	65 (34.9)
	Divorced	25 (13.4)
	Widowed	15 (8.1)
	Missing	8
Religious affiliation	Yes	76 (53.9)
	No	65 (46.1)
	Missing	53
Income	<$40,000	18 (9.4)
	$40,000–$75,000	138 (71.9)
	>$75,000	36 (18.8)
	Missing	2
History of mental health diagnosis	Yes	46 (23.7)
	No	148 (76.3)
Education level	High school or less	35 (48.6)
	College or higher	37 (51.4)
	Missing	122
Cancer type	Lung	69 (40.1)
	Head and neck cancers	30 (17.4)
	Other	73 (42.4)
	Missing	22
Referral acceptance	No	101 (52.1)
	Yes	93 (47.9)
Age	Median	60.50
	Minimum	21.00
	Maximum	84.00

*Note*: Cessation program by their cancer care provider between July 2019 and August 2021.

**FIGURE 3:**
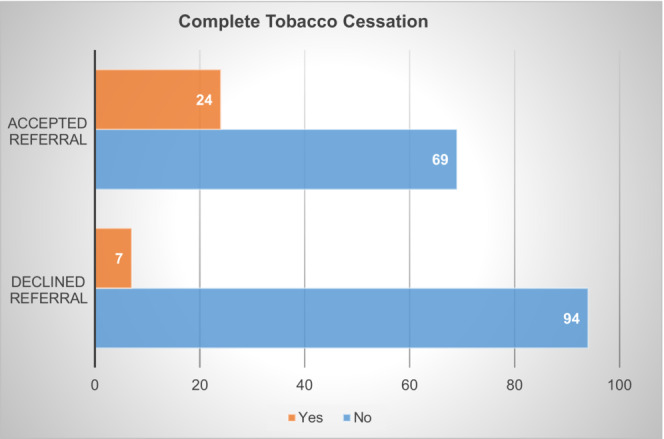
Rates of complete tobacco cessation. Those who accepted a referral to the tobacco cessation program had higher rates of complete tobacco cessation as compared to those who did not, which is statistically significant on the chi‐square analysis (*p* < 0.0001).

Overall, 24 patients out of the 93 patients (25.8%) engaging with cessation services had documentation of complete tobacco cessation. The majority of these patients (84, 90%) were initiated on pharmacotherapy. In comparison, only 7 of the 101 patients (6.9%) that did not engage in the program were able to cease tobacco use (*p* < 0.001) (Figure 3).

Initially, a univariate logistic regression was performed to look for any associations with referral acceptance. The Caucasian race (odds ratio [OR] = 0.51 [95% confidence interval, CI 0.28–0.93], *p* = 0.027) and marital status (overall *p* = 0.020) were the only covariates found to be statistically significant (Table 2). On multivariable analysis, using the backward elimination approach, race and marital status continued to be associated with referral acceptance (Table 3). Religious affiliation and education level were excluded from the analysis given the lack of data. After adjusting for other confounders, compared with married patients, single patients (OR = 0.33 [95% CI 0.16–0.69], *p* = 0.003), divorced patients (OR = 0.60 [95% CI 0.23–1.56], *p* = 0.3), and widowed patients (OR = 0.55 [95% CI 0.17–1.77], *p* = 0.32) were less likely to accept a referral. In addition, Caucasian patients (OR = 0.43 [95% CI (0.22–0.82)]) were less likely to accept a referral as compared with other races (*p* = 0.010).

**TABLE 2:** Univariate logistic regression assessing for correlation between referral acceptance and covariates

Covariate	Category	*N*	Odds ratio (95% CI)	OR *p* value
Gender	Male	106	0.61 (0.35–1.09)	0.094
	Female	88	‐	‐
Race	Caucasian	104	0.51 (0.28–0.93)	0.027*
	Non‐Caucasian	78	‐	‐
Insurance	Private	110	1.22 (0.56–2.65)	0.612
	Medicare	50	1.17 (0.49–2.81)	0.726
	Medicaid/uninsured	34	‐	‐
Marital status	Single	65	0.45 (0.23–0.88)	0.020*
	Divorced	25	0.67 (0.27–1.64)	0.379
	Widowed	15	0.83 (0.27–2.50)	0.736
	Married	81	‐	‐
Religious affiliation	Yes	76	1.23 (0.63–2.39)	0.541
	No	65	‐	‐
Income	<$40,000	18	1.25 (0.40–3.89)	0.701
	$40–75,000	138	0.86 (0.42–1.80)	0.698
	>$75,000	36	‐	‐
History of mental health diagnosis	Yes	46	1.11 (0.57–2.16)	0.748
	No	148	‐	‐
Education level	High school or less	35	0.89 (0.35–2.24)	0.803
	College or higher	37	‐	‐
Cancer type	Head and neck cancers	30	0.90 (0.38–2.13)	0.812
	Other	73	0.95 (0.49–1.83)	0.874
	Lung	69	‐	‐
Age		194	1.02 (1.00–1.04)	0.116

*Note*: An alpha of 0.2 was used for significance.

Abbreviations: CI, confidence interval; HNC, head and neck cancer; OR, odds ratio.

* *p* value is statistically significant.

**TABLE 3:** Multivariable logistic regression using the backward selection assessing for correlation between referral acceptance and covariates

Covariate	Category	*N*	Odds ratio (95% CI)	OR *p* value	Type 3 *p* value
Gender	Female	80	1.64 (0.88–3.08)	0.122	0.122
	Male	99	‐	‐	
Race	Caucasian	102	0.43 (0.22–0.82)	0.010	0.010*
	Non‐Caucasian	77	‐	‐	
Marital status	Single	62	0.33 (0.16–0.69)	0.003	0.033*
	Divorced	24	0.60 (0.23–1.56)	0.296	
	Widowed	15	0.55 (0.17–1.77)	0.318	
	Married	78	‐	‐	

*Note*: An alpha level of removal of 0.20 was used. The following covariates were removed from the model: age, cancer type, insurance type, median income of zip code, and mental health diagnosis.

Abbreviations: CI, confidence interval; OR, odds ratio.

* *p* value is statistically significant.

## 4 DISCUSSION

In this retrospective analysis of a single institution NCI‐designated Comprehensive Cancer Center, we found our cohort of cancer patients found that less than half of patients engaged with tobacco services when referred to a tobacco cessation program. Engagement of patients with the program (47%) was higher than reports from other NCI‐designated Comprehensive Cancer Centers (17% and 6.4%).^21^, ^22^ However, the referral process was initiated by the cancer care team provider (or a minority of patients who self‐referred), whereas other programs automatically referred a patient if tobacco use was documented in the electronic medical record, which precludes potential smokers from not being properly identified and referred by the provider. Therefore, it is not unexpected that the engagement rates are higher in our cohort.

We found unmarried and Caucasian patients were less likely to engage with cessation services. In an analysis of a cohort of cancer patients referred to a tobacco cessation program from another NCI‐designated Comprehensive Cancer Center, patients in a relationship and racial minorities were less likely to currently use tobacco. However, they found no correlation between race or relationship status with engagement with cessation services as we did.^21^ They did find males were less likely to accept a referral, though we did not find a correlation between gender and referral acceptance.

A report by the CDC on smoking rates by adults in the United States in 2019 found that the prevalence of current tobacco use was higher for males, single/divorced/widowed patients, and Caucasian patients compared with their counterparts.^2^ Perhaps, these subgroups may have greater exposure to tobacco products and increased nicotine dependence that drives the lower rate of referral acceptance among these groups. On univariate logistic regression, there was no association between referral acceptance and having a history of a mental health diagnosis. While mental health is certainly an important and intertwined issue in tobacco use, it did not clearly have an impact on a patient's likelihood to engage with the program in our cohort.

The tobacco cessation rate of 25.8% among patients that engaged with cessation services is comparable to other NCI‐designated Comprehensive Cancer Centers (15%–47%).^23^ Studies have shown certain subgroups of cancer patients have higher rates of tobacco cessation, such as lung cancer patients.^24^ In our cohort, 40% of patients were lung cancer patients. However, we did not find an association with lung cancer, though the sample size may not have been sufficiently powered to detect this. While the reviewer suggests lung cancer patients are harder to engage in cessation efforts, data indicate cancer patients with diagnoses related to tobacco use are more likely to quit using tobacco products after diagnosis.

In our cohort, 90% of patients that engaged with cessation services were initiated on pharmacotherapy. The National Comprehensive Cancer Network recommends a combination of behavioral therapy with pharmacotherapy for tobacco cessation.^25^ However, not all providers may feel equipped to counsel patients on tobacco cessation and the various treatment modalities available. A survey of over 1500 cancer providers found that while 90% of providers inquired if patients used tobacco products, only 40% discussed pharmacotherapy options.^26^ Given the effect of tobacco cessation on oncologic outcomes, it is imperative that patients who smoke are offered pharmacotherapy options.

Tobacco cessation is especially critical for cancer patients as continued tobacco use is associated with inferior oncologic outcomes and worse quality of life as well as increased risk of additional malignancies. Active engagement of cancer patients with tobacco cessation programs remains challenging. We found that only 48% of referred patients engaged with cessation services at our NCI‐designated Comprehensive Cancer Center.

For those patients that did engage with cessation services, the tobacco cessation rate was dramatically higher (25.8%) compared to the cessation rate of patients who did not engage (6.9%). We feel the convenience of a remote tobacco cessation specialist was a key part of the success. Many patients travel from rural areas and would be deterred by an additional drive to the city for a tobacco cessation consultation. The ability of tobacco specialists to facilitate pharmacotherapy initiation and refer patients to the Georgia quitline, which offers nicotine replacement at no cost, was also instrumental to the success of the program. Most patients that engaged with cessation services were initiated on pharmacotherapy (90%), underpinning the importance of pharmacotherapy in a successful tobacco cessation program. Unfortunately, studies have shown that many providers feel ill‐equipped to assist with tobacco cessation and initiation of pharmacotherapy, underscoring the importance of tobacco cessation programs that can counsel and prescribe pharmacotherapy.

Despite the success of the program, we recognize the limitations of our study. As previously indicated, tobacco cessation was not confirmed with biochemical verification. The tobacco cessation specialist documented if tobacco cessation was reported by the patient, which was captured in the chart reviews. Given the geographic spread of patients seen at our cancer center, many patients come from far away, and testing would be burdensome. There may be a slight discrepancy between the reported and actual tobacco cessation rates.

We further acknowledge that the missing data are a limitation. Demographic information, such as education level, religion, etc. is self‐reported by patients, and completion is not compulsory for office visits. While our sample size was not as large as a multi‐institutional study could have provided, it was large enough to identify an association between program engagement and the relationship status and race of patients. We did not evaluate if there was an association between the cancer stage or the intent of therapy (curative vs. palliative), which could potentially influence the motivation and willingness of a patient to engage with the program, though the analysis would have been limited given the sample size.

However, our study has its unique strengths. Although other cancer centers have published their experience with tobacco cessation programs, our experience is unique given the demographic spread of the participants. Often underrepresented in clinical trials, African Americans made up 38.7% of participants referred to the program. In comparison, only 18.3% of participants were African American in a tobacco cessation program at an NCI‐designated cancer center in Buffalo, NY, and only 6.08% of participants were African American at an NCI‐designated cancer center in Louisville, KY. We were able to identify that Caucasians were less likely to engage in the program compared with their non‐Caucasian counterparts, which is a finding that was only possible given the sizable amount of non‐Caucasian participants.

It is critical to develop novel interventions to increase the engagement of patients with tobacco cessation programs, especially unmarried and Caucasian populations as they were less likely to accept a referral to our tobacco cessation program. Cancer patients face distinct challenges in their tobacco cessation journey, such as feelings of being overwhelmed, and may not be properly counseled on the benefits of tobacco cessation on their oncologic outcomes and quality of life. Cancer providers are in a unique position to offer counseling and initiate pharmacotherapy at the time of a cancer diagnosis, during which there is often a high motivation to quit using tobacco products.

Tobacco cessation should not be neglected in cancer treatment but instead should be seen as important as other treatment modalities given the impact on survival and the quality of life. Our experience further adds to the body of literature that clearly illustrates the importance of tobacco cessation services and the need for innovation to generate novel ways to increase program engagement of populations that were identified as being less likely to engage. Better targeting and enrolling these patients is impactful as the rates of cessation services were dramatically higher in those that engaged in tobacco cessation services (25.8%) compared to those that did not (6.9%) in our cohort.

## AUTHOR CONTRIBUTIONS

**Manali Rupji:** Formal analysis (equal); methodology (equal); writing – review and editing (equal). **Lauren E. Franklin:** Conceptualization (equal); data curation (equal); methodology (equal); project administration (equal). **Madhusmita Behera:** Formal analysis (equal); supervision (equal); writing – review and editing (equal). **Suresh S. Ramalingam:** Conceptualization (equal); supervision (equal). **Kristin A. Higgins:** Conceptualization (equal); funding acquisition (equal); investigation (equal); methodology (equal); project administration (equal); resources (equal); supervision (equal); writing – review and editing (equal).

## CONFLICT OF INTEREST

The authors declare that they have no conflict of interest to report.

## Data Availability

The data that support the findings of this study are available from the corresponding author upon reasonable request.
